# Functionalized Graphene Oxide Modified Polyethersulfone Membranes for Low-Pressure Anionic Dye/Salt Fractionation

**DOI:** 10.3390/polym10070795

**Published:** 2018-07-19

**Authors:** Lifen Liu, Xin Xie, Rahul S. Zambare, Antony Prince James Selvaraj, Bhuvana NIL Sowrirajalu, Xiaoxiao Song, Chuyang Y. Tang, Congjie Gao

**Affiliations:** 1 Centre for Membrane and Water Science & Technology, Ocean College, Zhejiang University of Technology, Hangzhou 310014, China; lifenliu@zjut.edu.cn (L.L.); 2111601112@zjut.edu.cn (X.X.); gaocj@zjut.edu.cn (C.G.); 2 Environmental and Water Technology Centre of Innovation (EWTCOI), Ngee Ann Polytechnic, Singapore 599489, Singapore; rahul22288@gmail.com (R.S.Z.); bso2@np.edu.sg (B.N.S.); 3 Department of Civil Engineering, The University of Hong Kong, Pokfulam, Hong Kong, China; tangc@hku.hk

**Keywords:** polyethersulfone, graphene oxide, ionic liquid, polydopamine, nanofiltration, dye/salt fractionation

## Abstract

In this study, polyelectrolyte assembled functionalized graphene oxide (PE-GO) membranes were fabricated through a one-step charge facilitated deposition method for high performance dye/salt separation. According to the intercalation of polydopamine (PDA) and (ionic liquid) IL functional moieties into the GO membranes, the pore size of the resulted PE-pGO and PE-iGO membrane increased from 2.69 nm to 4.13 nm and 6.54 nm, respectively. Correspondingly, a pure water flux of 13.8 ± 2.2, 36.7 ± 3.4, and 52.1 ± 6.7 L m^−1^ h^−1^ bar^−1^ was achieved for PE-GO, PE-pGO and PE-iGO membrane, respectively. PE-iGO membrane with the largest pore size could be operated with significant water permeability (28.3 to 38.3 L m^−1^ h^−1^ bar^−1^) at a low operating pressure range of 0.5–2 bar (dye concentration = 100 ppm, salt concentration = 5 g/L). More importantly, functionalities introduced to the GO nanosheets are found to impact the dye adsorption to the membrane surface. The IL intercalation promotes the elution of dye molecules from the IL moieties at elevated pH, therefore enhancing the efficiency of alkaline washing of the membrane. By contrast, the intercalation of PDA weakens such efficiency due to its strong adhesion force to the dye molecules even at the alkaline condition.

## 1. Introduction

Membranes provide an efficient solution to treat dye industry wastewaters, which are usually produced with high salt and dye content [1]. The attempt for one-stop recovery of water from the wastewater faces challenges of high fouling propensity by high dye content and high energy requirement (to overcome osmotic pressure) for desalination membranes. Recently, nanofiltration has been proposed to solve the conundrum. Ideally, nanofiltration offers great opportunity to reject the dye molecules with high efficiency. Commercial nanofiltration membranes provides a flux of 1013 L m^−2^ h^−1^ bar^−1^ [2]. However, the nanofiltration tends to reject both anionic dyes and salts, thus the need to overcome osmotic pressure generated by salts intensifies the energy requirement [3]. Typically, commercial nanofiltration (NF) and reverse osmosis (RO) membranes pressure of 1020 bar to achieve a flux of 30–60 L m^−2^ h^−1^ [4]. Moreover, the high pressure required for NF operation entails higher external concentration polarization and irreversible fouling tendency by the dye molecules [3,5]. Ultrafiltration (UF) process is an alternative process for the treatment of dye wastewater with enhanced flux, however, the low rejection efficiency limits the widespread use of UF membrane in dye industry and the fouling tendency (due to physical adsorption, pore blocking and cake formation) lead to severe compromise of water flux [5,6,7]. Therefore, there is a “balance” zone between NF and UF membranes, namely loose NF/tight UF, which carries high rejection for dye molecules yet low retention to salts rejection. 

Recently, GO and rGO membranes are gaining popularity for small molecules selective sieving because of the size exclusion and charge repulsion in the inherent nanoscale water channels confined between graphene basal planes [8]. The GO and rGO membranes have been frequently assembled using vacuum filtration, which is typically a time-consuming lab-scale process and difficult to scale up [9]. In addition, the as-assembled membranes usually face stability issues in aqueous solution [10,11]. Therefore, the overall cost and assembling technique need to be improved in favor of the mass production of GO/rGO membranes in liquid filtration. In addition, the vacuum-assembled GO and rGO membranes tend to reject monovalent and divalent salts quite efficiently, which incurs higher operation pressure to offset the partial osmotic pressure [7].

A preferred nanofiltration membrane for dye wastewater treatment should possess advantages such as high rejection of dye molecules, low retention of salt, high hydrophilicity, durability and resistance to irreversible fouling [5]. In recent years, few-layered GO gated membranes offer great opportunities for ultrafast molecular-separation membranes through GO channels [12,13]. Reducing the number of GO layers greatly enhances the water permeability due to less resistance [14]. Therefore, surface functioning by a few layers of GO appears to be the right formula for dye filtration membranes. To date, the molecular weight cut-off (MWCO) of these membranes, a metric for membrane’s (in the loose NF/tight UF range) sieving ability towards neutral molecules, is yet to be understood and related to the performance of such membranes. Furthermore, surface modification of GO nanosheets impacts its interaction with the dye molecules at the membrane–water interface. Enlightened by this, hydrophilic PDA modification [15] and pH-responsive IL grafting [16] are proposed in the current paper to mitigate the irreversible fouling of dyes by providing a customizable low/reversible adsorption surface. Herein, we propose an approach of assembling the functionalized GO as the rejection layers by one-step electrostatic deposition. MWCO, pore size distribution, and fouling propensities of the as-formed membranes were systematically investigated during the dye/salt mixture filtration experiment.

## 2. Materials and Methods 

### 2.1. Chemicals and Reagents

Dopamine hydrochloride (98%) powder, Tris-HCl buffer, 1-Ethyl-3-(3-dimethylaminopropyl) carbodiimide hydrochloride (EDCI), *N*-hydroxy-succinimide (NHS), Poly(allylamine hydrochloride) (PAH, M_w_ ~50 kDa), Poly(sodium 4-styrene-sulfonate) (PSS, M_w_ ~70 kDa), direct red 80 (DR80), Congo red (CR), and Polyethylene Glycol (PEG) with different molecular weights were purchased from Aladdin, Shanghai, China. 1-Methylimidazole and 3-bromopropylamine hydrobromide (98%) were purchased from Energy Chemicals, Shanghai, China. Solvents were purchased from Sinopharm, Shanghai, China.

### 2.2. Synthesis of GO, iGO, and pGO Nanosheets

GO nanosheets were synthesized according to a modified Hummer’s method according to our previous reports [16,17]. Subsequently, iGO nanosheets were formed by binding methylimidazolium ionic liquid (IL) with carboxylic groups on GO, mediated by EDCI and NHS. Briefly, amino-terminated IL was formed by reaction and crystallization of 1-Methylimidazole and 3-bromopropylamine hydrobromide (1:1 molar ratio) in N_2_ atmosphere [16]. Subsequently, amino-terminated IL was grafted onto GO by stirring 3 mmol of EDCI and NHS together with 30 mg of GO dispersion in DMF at 0 °C for 2 h, adding 3 mmol of amino-terminated IL, and reacting at room temperature for another 22 h. The iGO was isolated from the mixture, washed, and free-dried. PDA−GO (pGO) nanosheets were synthesized by in-situ polymerization of polydopamine (PDA) on GO nanosheets [18]. Briefly, 10 mg of GO were dispersed in 50 mL Tris-HCl buffer solution (10 mmol/L, pH 8.5) in an ultrasound bath (Elmasonic E60H) for 1 h. Then, 100 mg of dopamine were added to the mixture and the GO mediated polymerization of dopamine was continued for 24 h at 60 °C. The pGO was separated by centrifuge, washed, free-dried, and stored for further usage.

### 2.3. Membrane Fabrication

The UH 030 substrate flat sheet membranes (polyethersulfone), denoted as UF30, were kindly provided by Microdyn-Nadir (Xiamen, China) with nominal MWCO of 30 kDa. Substrate membranes were used without further treatment. Polyelectrolytes deposition solutions were prepared by dissolving 1000 ppm PAH or PSS in 0.5 mol/L NaCl solution as described in our previous study [19]. Membrane coupons, rinsed with sufficient DI water, was first treated with PSS saline solution for 10 min. Subsequently, the PSS deposited UF membranes were rinsed with DI water for 5 min and treated with PAH saline solution for another 10 min. The formed membrane surface has 1 bilayer (BL) of PSS/PAH terminated with a positive charge. Finally, the modified membranes were immersed in 0.1 g/L GO, pGO, or iGO aqueous solutions for 6 h for the absorption of GO-based nanosheets. The GO, pGO, or iGO coated composite membranes, namely PE-GO, PE-pGO, and PE-iGO membranes, are then rinsed with and kept in DI water below 5 °C. 

### 2.4. Pore Size Evaluation of the GO-Based Membranes

The PEG rejection test was employed to characterize the pore size distribution of the GO-based membranes. The Stokes radii of the solutes can be related to average molecular weights using an empirical equation [20]. For PEG:

(1) $$r=16.73\times 10^{-12}\times M^{0.557},$$

where *r* is the Stokes radii of the molecule (*m*) and *M* is the molecular weight (g/mol). In theory, the rejection towards a solute can be expressed as the integration of the log-normal distribution probability density function [21,22]:

(2) $$R=\mathrm{erf}\left(y\right)=\frac{1}{\sqrt{2\pi }}\int_{-\infty }^{y}e^{-u^{2}/2}du,\;\mathrm{where}\;y=\frac{\ln r_{s}-\ln\bar{r_{s}}}{\ln\sigma_{g}}\;,$$

where $r_{s}$ is the radius of the solute, $\bar{r_{s}}$ is the geometric mean of the solute radius when *R* = 50%, $\sigma_{g}$ can be determined by the ratio of $r_{s}$ at *R* = 84.13% and *R* = 50%. Correspondingly, on a log-normal probability coordinates, the plotted curve of R and $r_{s}$ should linearize in the form of:

(3) $$F\left(R\right)=A+B\times \ln r_{s},$$

The fitted curve can derivate the values of $\bar{r_{s}}$ and $\sigma_{g}$ and thus the probability density function (PDF) and cumulative PDF for the corresponding membrane can be determined. 

### 2.5. Characterization of the PE-GO Membranes

Zeta potential and particle size of the GO/iGO/pGO nanosheets were determined using a Brookhaven Omni unit (Holtsville, NY, USA), in PALS and DLS mode, respectively. TEM images for GO/iGO/pGO nanosheets were obtained by a JEOL JEM-2100 unit (JEOL, Tokyo, Japan). For the Chemical structure of both GO powder and membrane, samples were characterized by FTIR spectra (Shimadzu FTIR-8400S, Kyoto, Japan). Surface Zeta potential (ζ) of PE-GO membranes was tested with an Anton-Paar Surpass^TM^ 3 Unit (Anton-Paar, Graz, Austria). Surface and cross-section morphology of the membrane coupons were observed with an ultrahigh-resolution Hitachi 8010U FESEM unit (Kyoto, Japan). AFM images for membrane samples were taken on a Bruker unit (ICON, Peak-force Mode) (Billerica, MA, USA). The hydrophilicity of the surface was characterized by the contact angle measurement (Dataphysics, OCA 50, Filderstadt, Germany).

### 2.6. Performance Evaluation of the Membranes

**Figure 1:**
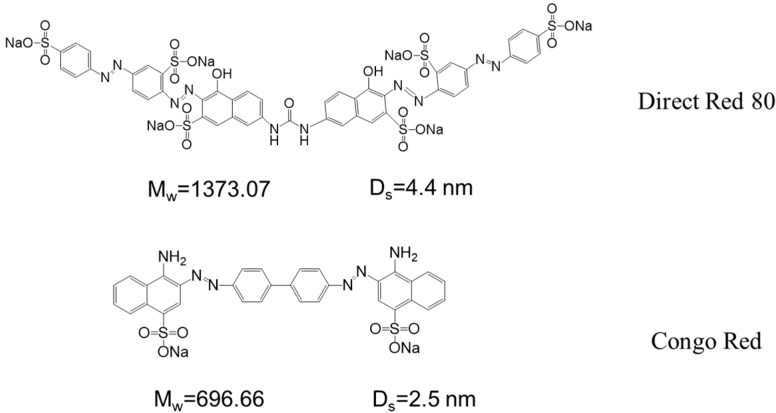
The molecular structure, molecular weight, and estimated molecular size of DR80 and CR. Molecular size was estimated according to a previous study [17].

A commercial Merch Millipore (Burlington, MA, USA) 8010 cell was used to evaluate the effect of dye concentration and operating pressure on the transmembrane flux and dye rejection rate. Unless specified, the dye concentration was 100 ppm and operating pressure was at 2 bar. The MWCO, saline dye wastewater fractionation, and long-term performance test was carried out using a homemade cross-flow setup with an effective membrane area of 8 cm^2^ (4 cm × 2 cm). The cross-flow rate was kept constantly at 20 cm/s for all measurements. Flux was calculated based on the time required to collect 15 mL of filtrate. The dye rejection was calculated based on the following equation: R = 1 − Cp/Cf. Anionic dyes with different molecular weight, namely direct red 80 (DR80) and Congo red (CR), were used as model dyes. Their molecular structure, molecular weight, and estimated molecular size are summarized in Figure 1.

## 3. Results and Discussions

### 3.1. Characterization of GO, pGO, iGO Nanosheets and PE-GO Membranes

**Figure 2:**
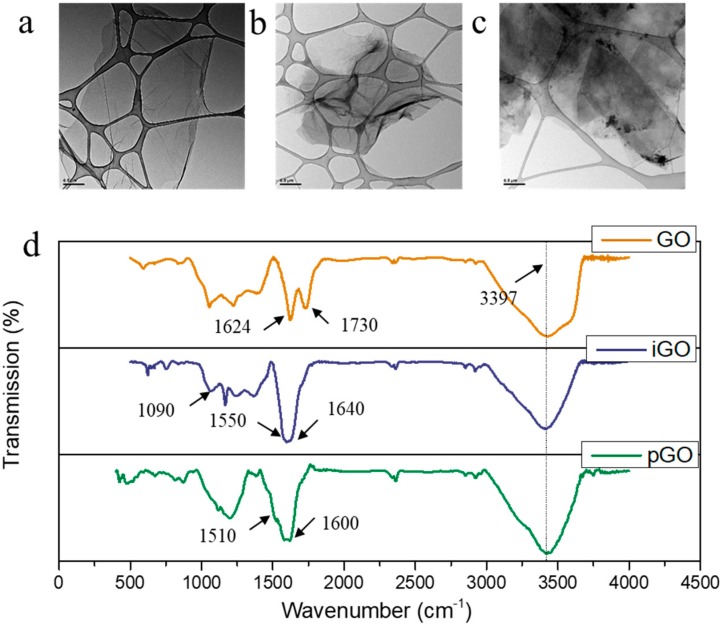
The TEM images of nanosheets: as-synthesized (**a**); GO (**b**) iGO; and (**c**) pGO. The FTIR spectrum of the nanosheets (**d**).

As the synthesis of GO, pGO, and iGO has been comparatively well studied in some earlier published results, we briefly characterized them using TEM, FTIR, and DLS characterization. GO, iGO, and pGO dispersions show goldish, yellowish brown and dark brown colors, respectively (Figure S1). TEM Micro-images show the isolated GO, iGO, pGO nanosheets from the aqueous solutions are mainly dispersed nanosheets (Figure 2a–c). The average size of pGO nanosheets (Table 1) is slightly small than the iGO and pristine GO nanosheets. We infer that it might be associated with the reduction by PDA [18]. The FTIR results confirmed the successful synthesis of GO. The peaks at 3397 cm^−1^, 1730 cm^−1^, and 1624 cm^−1^ are assigned to υ (O–H), υ (C=O), and υ (C=C) stretching mode, respectively [17]. The successful grafting of the ionic liquid brought about new absorption peaks near 1640 cm^−1^, 1550 cm^−1^, and 1090 cm^−1^, attributed to amide-carboxyl, υ (N–H), and υ (C–N) in stretching mode [16]. The prominent υ (O–H) peak remains at 3397 cm^−1^, suggesting the hydroxyl groups were maintained. For pGO, however, the new peaks at 1600 cm^−1^ and 1510 cm^−1^ are attributed to υ (C=C) resonance mode and υ (N–H) bending mode, respectively [23]. Interestingly, the strength of the υ (O–H) absorption band weakened, possibly due to the partial reduction of GO by pDA [18]. However, the GO, pGO, and iGO coated membranes showed no apparent difference in ATR-FTIR (Figure S2). This is probably because of the thin GO layer assembled on the top layer is too thin to result in significant absorption bands considering the penetration depth of IR in the range of microns [24].

**Figure 3:**
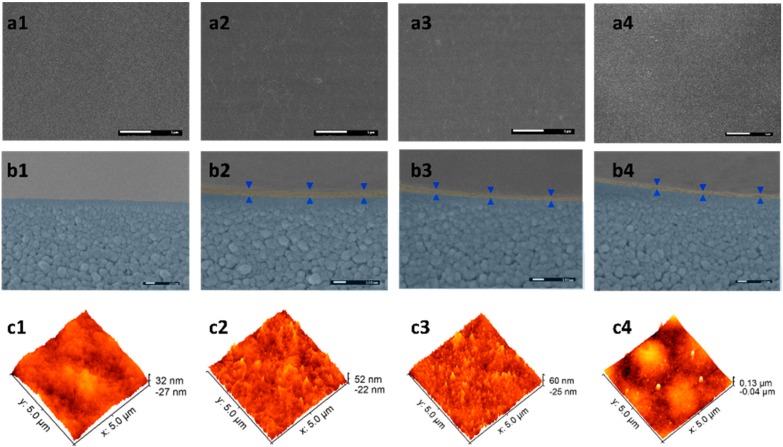
(**a1**–**a4**) The FESEM surface images of the UF30, PE-GO, PE-pGO, and PE-iGO membranes. The length of scale bar (white) is 1 µm; (**b1**–**b4**) The cross-section images of the same membranes. The rejective layer is highlighted with shallow orange color. The length of scale bar (white) is 100 nm; (**c1**–**c4**) AFM scans on the surface of the same membranes.

FESEM, AFM, zeta potential, contact angle, and FTIR were used to systematically characterize the morphology and hydrophilicity of the PE-GO membranes. The plain UF30 surface is rather smooth and characterized by nodule-like (in the nano-range) structures (Figure 3(a1)), which is usually formed on polyethersulfone-based membranes fabricated via phase separation method [25]. Pores on the surface could not be observed due to the relatively small MWCO of this membrane. Such smooth surface features an RMS of 7.9 nm (Figure 3(c1)). The contact angle of this membrane was 58.6 ± 4.9°. After electrostatic deposition of the GO nanosheets to the surface, it shows a typical wrinkled surface of graphene oxide; nonetheless, the overall roughness was not increased significantly (7.7 nm). The deposition of pGO nanosheets, however, resulted in an increased RMS of 13.3 nm. The increment can be explained by the in-situ synthesized PDA particles on pGO nanosheets [26]. When iGO nanosheets are deposited, the resultant membrane surface showed a slight increase of roughness of 8.1 nm, which could be due to the grafting of IL onto GO nanosheets, increasing the intrinsic roughness of the GO nanosheets [16,17]. As the negatively charged GO nanosheets exhibit macromolecular behaviors in aqueous solution [27], as-formed PE-GO membranes resemble the layer-by-layer formation of polyelectrolyte membranes. As it is electrostatically bonded at the molecular level, it could endure harsh perturbations, such as ultrasonication, without observable detachment. From the cross-section view of the micro-images (Figure 3(b1–b4)), the deposition of modified GO nanosheets created a different thickness of the functional layer, which is distinctive from the nodular-like porous support membrane. The top layer thickness (δ), which is indicated by the distance between the opposingly-placed triangles, follows the order of δ_GO_ (57.0 ± 3.6 nm) > δ_pGO_ (45.4 ± 5.5 nm) > δ_iGO_ (34.0 ± 4.7 nm). 

**Figure 4:**
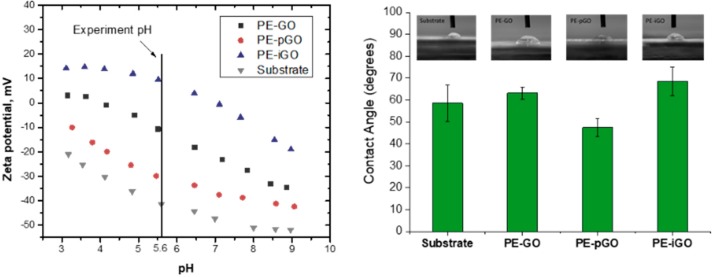
(**a**) The Surface Zeta potential of PE-GO, PE-pGO, PE-iGO, and the substrate membrane as a function of pH value; and (**b**) the contact angle of the said membranes; insert pictures shows water droplets on top of the same membranes.

At experimental pH, the surface zeta potential responsivity was −10.69 ± 2.4, −29.9 ± 1.5, and 9.5 ± 0.6 mV for PE-GO, PE-pGO, and PE-iGO membranes, respectively (Figure 4). Note that the zeta potential is highly related with the charge of ζ of the GO/modified GO suspensions (ζ_GO_ = −34.3 ± 2.2 mV, ζ_pGO_ = −37.1 ± 1.1 mV, and ζ_iGO_ = 11.4 ± 0.6 mV, as shown in Table 1). 

This suggests the deposited GO nanosheets completely altered the surface charged of the PAH intermediate layer. This phenomenon agrees well with the layer-by-layer process where the surface charge oscillates according to the alternative deposition of positively/negatively polyelectrolytes [19]. Nonetheless, the absolute value of the zeta potential is less than that of its free dispersion. Likely due to a close packing of GO nanosheets, this phenomenon has also been observed in our previous study on amine-functionalized GO [17]. Note that the PE-pGO possesses the most negatively charged surface, leading to a more hydrophilic surface with the lowest contact angle of 47.4 ± 2.3° (Figure 4) [28]. In contrast, the PE-GO and PE-iGO membranes have an elevated contact angle of 63.1 ± 1.5° and 68.5 ± 5.1°, respectively (compared with 58.6 ± 4.9° of the support membrane). This might be contributed by the decreased pore size or partially contributed by the hydrophobic PAH molecules in the intermediate layer [19].

### 3.2. Pore Size Characterization of the PE-GO Membranes

**Figure 5:**
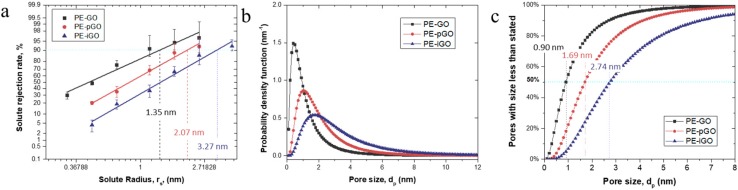
(**a**) The log-normal plot of solute rejection rate versus solute radius for PE-GO, PE-pGO, and PE-iGO membranes; (**b**) the probability density function curve of pore radius for the membranes; and (**c**) the cumulative pore distribution curve of pore radius for the membranes.

Series of PEG molecules were filtrated through the membrane to test the MWCO of the PE-GO membranes. We adopted the model developed by Michaels, in which the log-normal probability distribution of rejection is linearly fitted with the Stokes radii, *r*, equivalent to the MWCO values of the membranes [21]. Figure 5 shows the log-normal plot of the rejection of solutes versus their corresponding rejections, which can be relatively well fitted with the linear relationship. Towards the same grade of PEG, the PE-GO showed the highest rejection for PEG, while the PE-iGO showed the lowest rejection (Figure 5a). This trend can be explained by the relatively thinner active layer of PE-iGO membrane, likely due to the unfavored electrostatic deposition of positively charged iGO nanosheets to the PAH intermediate layer. Moreover, the PE-iGO have a larger d-spacing due to the intercalation of IL [16], thus allowing larger PEG molecules to pass through. The MWCO values of the PE-GO, PE-pGO, and PE-iGO membranes can be derived from the probability/cumulative density function (at 90% cut-off point) to be 2632, 5685, and 12,989, respectively, corresponding to pore sizes of 2.70, 4.14, and 6.54 nm (Figure 5a). Note that the median pore diameters of the membranes are 0.90, 1.69, and 2.74 nm, respectively (Figure 5b,c). The deviations of the pore sizes indicate that the size distribution of the as-assembled GO membranes is far from the perfect layered structure, but rather containing some larger defects due to edge effect [29], aggregation [30], or intrinsic defects [31]. Nonetheless, the differentiated pore size distribution of PE-GO membranes has been achieved by manipulating the charge/functional groups on GO nanosheets. This approach provides an opportunity to customize the pore size of the resultant membrane in a few nanometers, which is ideal for the high-performance separation of dye molecules [32,33].

### 3.3. Performance Evaluation of the PE-GO Membranes

**Figure 6:**
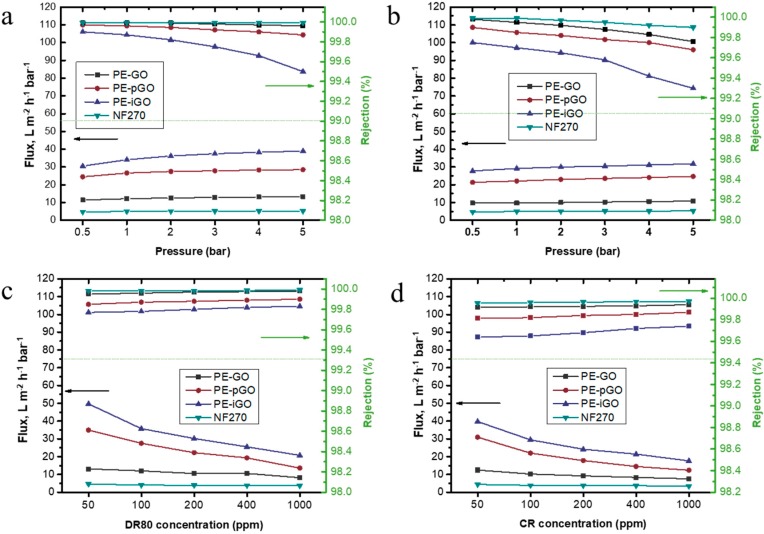
(**a**,**b**) The impact of operation pressure on the DR80 and CR rejection and flux evaluated using dead-end flow configuration. Stirring speed = 600 rpm. Dye concentration = 100 ppm; (**c**,**d**) The impact of DR80 and CR concentration on the flux and rejection evaluated using dead-end flow configuration. Operation pressure = 2 bar, stirring speed = 600 rpm.

The properties of the PE-GO membranes are summarized in Table 2. Prior to Cross-flow test, the effect of dye concentration and applied pressure on the membrane flux and dye rejection are investigated using a dead-end testing cell. In the study, two commonly used anionic dyes were adopted, DR 80 and CR. As a comparison, commercial NF270 was evaluated under the same conditions. In Figure 6a, the rejection of DR80 was tested under different operation pressure. Overall, the rejection of the membranes followed the order: R_NF270_ > R_PE-GO_ > R_PE-pGO_ > R_PE-iGO_. All membranes achieved a rejection of >99.5%. These rejection values are roughly 1–2 orders of magnitude higher than UF30, UF100, and UF150 ultrafiltration membranes (Figure S3). At such high rejection rate, the filtrate was almost colorless (see Figure S3). As the MWCO value of NF270 was reported to be 400 Da [34], it is generally concluded that (among the membranes tested), as the MWCO value of the membrane decreases, the rejection of the dyes increases. It is worth noting that the PE-pGO and PE-iGO membranes, even though they have much larger pore size than the size of dye molecules, achieved fairly high rejection towards the anionic dyes thanks to the Donnan exclusion effect [35].

Although the pure water permeability of NF270 was reported to be around 15 L m^−2^ h^−1^ bar^−1^ [36], the observed flux in this study was only ~5 L m^−2^ h^−1^ bar^−1^. This probably resulted from the continuous deposition of dye molecules at low cross-flow conditions in the dead-end configuration. As a comparison, the flux achieved in DR80 filtration by PE-GO, PE-pGO, and PE-iGO membrane was approximately 1.5, 4, and 6 times higher than that of NF270 membrane (Figure 6a). As the increase of the applied pressure, the flux (per unit of pressure) slightly increased for both of DR80 and CR (Figure 6a,b). This is because the effective pressure is lower than the apparent pressure due to the resistance contributed by the dye molecules deposited on the membrane surface. As the applied pressure was increased, while the dye concentration was not changed, the effective fraction of pressure increased, leading to an increased observed unit flux. At the same time, the rejection of dyes slightly decreased as the increase of pressure, especially for the PE-iGO membrane (Figure 6a). Note that the pore size of the PE-iGO (6.54 nm) is larger than DR80 (~4.4 nm). Although the comparatively larger size of the pores has been compensated by the Donnan exclusion effect, the higher convection at higher applied pressure seems to result in lower rejection. In this regard, the operating at low pressure is beneficial for a higher quality effluent. Out of a similar reason, the rejection was observed to decrease faster at higher operating pressure for even smaller CR molecules (~2.5 nm, Figure 6b). 

When the dye concentration increases, the flux generally decreases (Figure 6c,d). Note that, in dead-end configuration, the resistance is proportionally increased with the rejected dye molecules. For example, the flux for PE-GO, PE-pGO, PE-iGO, and NF270 membrane was reduced to approximately 62%, 39%, 42%, and 80% of its respective original value during DR80 filtration (Figure 6c). This is because the higher J_w_ induced quicker deposition of dye molecules on membranes, resulting in a more significant decline of the flux. Maintaining a low flux, especially lower than the critical flux [37], is beneficial for the sustainable operation. In addition, the test shows the capability for the PE-GO membranes to run at high recovery rates. When the feed solution became concentrated, the membranes appeared to have higher DR80 and CR rejection (Figure 6c,d), which was likely due to the lower convection flow induced by lower transmembrane flux. 

### 3.4. Saline Dye Wastewater Fractionation Simulation

**Figure 7:**
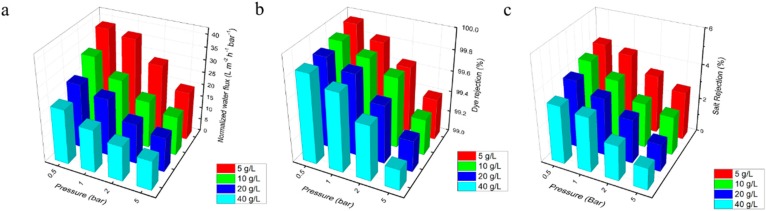
The (**a**) normalized water flux, (**b**) dye rejection, and (**c**) salt rejection for IL-GO membrane under different pressure and salt concentrations, DR80 concentration = 100 ppm. Pressure = 2 bar. The test was carried out in cross-flow configuration.

To simulate the real saline dye wastewater scenario, we have tested the salt/dye fractionation performance of the PE-GO membranes with varied operating pressure (0.5–5 bar) and salt concentration (5–40 g/L). In each run, the membrane was operated for 60 min and the water flux, dye rejection, and salt rejection were recorded. PE-iGO membrane was used as the outstanding example as it has the highest flux among the PE-GO membranes. It can be seen in Figure 7a that salt concentration impacts the water flux significantly. At 0.5 bar of pressure, 5 g/L salt resulted in 38.4 L m^−2^ h^−1^ bar^−1^. When the salt content was increased to 40 g/L, water flux decreased to 22.3 L m^−2^ h^−1^ bar^−1^ (i.e. ~42% in reduction). At a high pressure of 5 bar, the reduction was from 19.8 L m^−2^ h^−1^ bar^−1^ to 12 L m^−2^ h^−1^ bar^−1^ (i.e., ~39% in reduction). The reduction of water flux at higher salt content, likely due to the aggregation of destabilized dye molecules, has also been reported in the literature [32]. It seems to be a contradiction to the dead-end test that the unit flux decreased with increased pressure in the cross-flow test. A reasonable explanation is a difference in the filtrate volume. In the cross-flow test, the membrane has been operated for much longer than the dead-end cell, hence more solid has been deposited on the membrane surface, resulting in significantly reduced unit water flux. 

The dye rejection rate, on the other hand, was impacted less by the salt concentration at low operating pressure compared with high operating pressure. At 0.5 bar, the dye rejection dropped slightly from 99.95% to 99.85% as salt concentration increased from 5 g/L to 40 g/L. At 5 bar, the dye rejection dropped from 99.4% to 99.2%. This was because the electrostatic double layer effects were shielded [38] and the effective pore size was increased at higher salt concentration [35], resulting in deteriorated rejection, especially at higher convective flux. As the comparison, the operation pressure had a greater effect on the dye rejection. As analyzed before, the pore size of the PE-iGO membrane (6.54 nm) is much larger than the DR80 molecules (~4.4 nm), thus the Donnan exclusion effect is crucial to maintaining high rejection performance. When the Donnan exclusion effect is weakened by the background ionic strength of high salt concentration, one compensation solution is to reduce the operating pressure.

The salt rejection follows the same order with the dye rejection (Figure 7c), as the effect of Donnan exclusion on the charged salt ions resembles that on the charged dye molecules in. However, as the hydrated salt ions are significantly smaller, the salt rejection is roughly one order of magnitude lower than the dye rejection, suggesting a much weaker Donnan effect. Generally, the NaCl was rejected at a rate of 1–5%.

### 3.5. Stability Test of GO-Based Membranes

**Figure 8:**
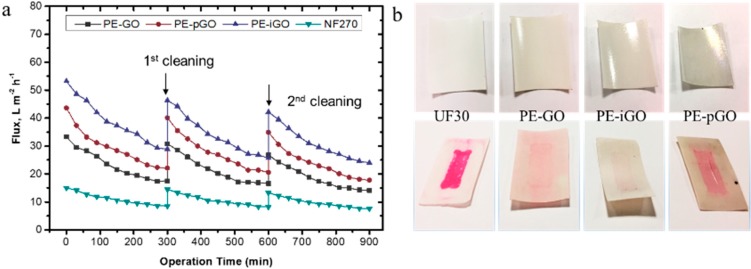
(**a**) The long-term fouling recovery test for PE-GO membranes. DR80 concentration = 100 ppm. Pressure = 2 bar. The test was carried out in cross-flow configuration. (**b**) The digital photos show a comparison of virgin membranes versus the fouled membranes (after alkaline cleaning).

The long-term performance of GO-based membranes was evaluated in the current study. The alkaline cleaning process was done after membrane operated for 300 min (Salt = 5 g/L, dye = 100 ppm, pressure = 2 bar). As a comparison, the commercial NF270 membrane was evaluated under identical conditions. As shown in Figure 8, significantly higher initial flux was shown for PE-iGO membrane (53.2 L m^−2^ h^−1^ bar^−1^) compared with the NF270 (~15.0 L m^−2^ h^−1^ bar^−1^). As the test was carried on, dye molecules started to deposit on the membrane surface, resulting in a slowly declining flux over 300 min when approximately half of the initial flux was achieved (29.3 L m^−2^ h^−1^ bar^−1^ for PE-iGO and 8.6 L m^−2^ h^−1^ bar^−1^ for NF270). During this process, the PE-iGO maintained a flux approximately two times higher than the NF270 membrane. After alkaline washing, the flux could be recovered to 87% of its initial value (91% in the 2nd cycle). As a comparison, the flux recovery rate for NF270 was 96.5% in the 1st cycle and 92% in the 2nd cycle. 

**Figure 9:**
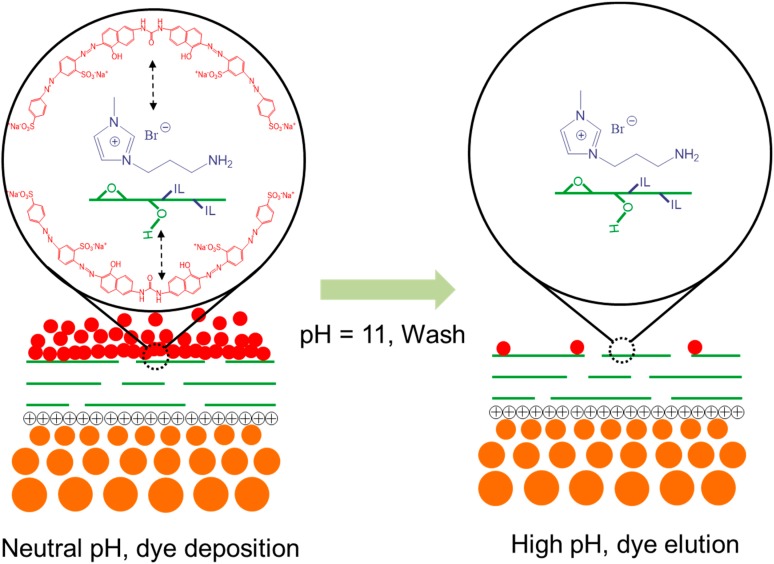
An illustration shows the deposition of DR80 molecules on PE-iGO membrane during dye filtration process (**left**) and the elution of dye molecules during alkaline wash due to weakened absorption force (**right**) (pH = 11).

More importantly, the alkaline cleaning was found more efficient for PE-GO membranes, especially PE-GO and PE-iGO membrane. As can be observed in Figure 8b, the UF30 shows a comparative example of irreversible fouling by deposited dye molecules. This is because of: (a) the adsorption of dyes (especially under high ionic strength conditions [32]) to hydrophobic polysulfone; and (b) the penetration of small dye molecules into the UF membrane substrate, leading to irreversible fouling by dye molecules that could not be recovered under alkaline conditions. By contrast, the washed surface of PE-GO membrane surface shows a much shallower color, which probably resulted from: (a) the GO rejection layer inhibiting the penetration of dye molecules; and (b) the hydrophilicity modification by GO nanosheets providing a hydrophilic surface with a protection layer of water molecules, which weakens the hydrophobic interactions of the dye molecules. When the solution condition was changed to alkaline during washing, the solubility of the DR80 increases and could be cleaned more efficiently. Unexpectedly, the dissociation of dye molecules on the more hydrophilic PE-pGO membrane surface appeared to be less favorable than the PE-GO membrane. This is probably because the PDA molecules strongly adhere to the dye molecules through the electrostatic, hydrogen bonds, and hydrophobic interactions [15,39], which becomes unstable only in strongly alkaline conditions (e.g., pH = 13) [40,41]. The PE-iGO surface appeared to be the least marked by the dye molecules. This phenomenon can be explained by the adsorption (filtration)–desorption (cleaning) mechanism at the interface between the membrane and dye molecules (Figure 9). Due to the fast interaction between iGO and dye molecules, the PE-iGO membrane surface should form a pre-adsorbed layer of dye molecules which inhibits subsequent dye molecules from entering the pores. At the cleaning stage, however, the adhesion force of the pre-adsorbed layer of dye molecules was reduced because of the deprotonation of the imidazolium cations and weakened electrostatic adsorption [16], resulting in the elution of DR80 and the regeneration of the IL-GO membrane. 

In conclusion, the inter-molecular interactions are crucial for the surface anti-adhesion properties of dye separation membranes. Hydrophilicity is the favorable property of such membranes. The grafting of pH-responsive materials, such as ionic liquid, adds another benefit as the cleaning/recovery of the membrane can be improved. In Table 3, we briefly summarize the performance of the loose nanofiltration/tight ultrafiltration membranes so far for the dye separation. The PE-iGO membrane is among the highest performance membranes thanks to the fine-tuned balance of permeability and selectivity. More importantly, the pH-responsive selective layer resulted in a low affinity to dye molecules in elevated pH, which favors the membrane regeneration in the alkaline conditions. Finally, the PE-iGO membrane favors the low-pressure filtration, which not only offers versatility in membrane configuration (e.g., submerged, vacuum suction, and hollow fiber) but also reduces the chance of ECP, dye penetration, and fouling.

## 4. Conclusions

Our work reveals that electrostatic deposition of functionalized GO nanosheets is a versatile method of forming a range of tight UF/loose NF membranes (with variable pore sizes) for high-performance dye/salt mixture fractionation. The resultant loose PE-GO nanofiltration membranes, with the pore size of a few nanometers, showed near perfect rejection (i.e., >99.5%) towards anionic dyes. Particularly, the PE-iGO membrane can be operated at a low operating pressure of 0.5 bar while achieving a high permeability of ~38.4 L m^−2^ h^−1^ (100 ppm DR80, 5 g/L NaCl), and the salt rejection for 10 g/L NaCl was constantly lower than 5%. Charge exclusion mechanism is the main mechanism attributing to such high rejection towards anionic dyes, considering the MWCO of the PE-GO membranes are significantly larger than typical NF membranes. Despite the advantages, the GO deposition techniques need to be further investigated in future work to shorten the immersion time, thus to increase the scalability for the PE-GO membranes. 

## Figures and Tables

**Table 1:** The physical size and ζ measurements for GO, iGO, and pGO, as measured in DLS and PALS mode.

Type	Size (nm)	Zeta potential (mV)
GO	528.0 ± 33.6	−34.3 ± 2.2
pGO	451.8 ± 25.9	−37.1 ± 1.1
iGO	493.3 ± 55.1	11.4 ± 0.6

**Table 2:** The Properties of PE-GO membranes.

Membrane	PWP (L m^−2^ h^−1^ bar^−1^)	WCA (degree)	RMS (nm)	SAD (%)	t (nm)	Pore size (nm)	MWCO (Da)
Substrate	206.1 ± 28.9	58.6 ± 4.9	7.9	16.2	−	8.3 ^a^	20,000 ^b^
PE-GO	13.8 ± 2.2	63.1 ± 1.5	7.7	19.0	57.0 ± 3.6	2.7 ± 0.5	2632 ^c^
PE-pGO	36.7 ± 3.4	47.4 ± 2.3	13.3	17.1	45.4 ± 5.5	4.1 ± 1.4	5685 ^c^
PE-iGO	52.1 ± 6.7	68.5 ± 5.1	8.1	23.1	34.0 ± 4.7	6.5 ± 1.6	12,989 ^c^

Note: ^a^ Pore size is calculated by Equation (1) using the reported nominal MWCO of 20,000 Da; ^b^ MWCO is reported by membrane manufacturer; ^c^ Values are calculated by Equation (1) using average pore sizes.

**Table 3:** Performance comparison of PE-GO membranes with other representative loose NF/tight UF membranes reported in the literature for dye separation.

Membrane	Dye filtration flux (L m^−2^ h^−1^ bar^−1^)	Operation pressure (MPa)	Dye conc. (g/L)	Dye	Salt conc. (g/L)	Dye rejection (%)	Salt rejection (%)	Ref.
RGO-CNT	10.11	0.5	~0.02	Direct yellow	0.6	99.6	39.6 ^a^	[42]
GO-PSBMA	9.4	0.4	0.5	Reactive black 5	1	99.2	<10 ^a^	[43]
PES/zwitterion-hydrotalcite	14.1 ^b^	0.4	0.5	Reactive red 49	1	90.3	0.36	[44]
PAEK-COOH	27.5	0.4	0.1	Congo red	0	95	-	[33]
Sepro NF 6	14.0	0.6	0.1	DR80	5	99.95	12	[3]
UH004	27.0	0.6	2	DR80	0–60	>98.9	2.6% ^c^	[32]
PE-iGO	28.3	0.2	0.1	DR80	5	99.65	<10	This work

^a^ Salt rejection measured separated from the dye solution; ^b^ Only pure water flux was reported; ^c^ Salt used was Na_2_SO_4_.

## Supplementary Materials

The following are available online at http://www.mdpi.com/2073-4360/10/7/795/s1, Figure S1: The as synthesized GO, iGO and, pGO aqueous solution, Figure S2: The ATR-FTIR absorption bands for UF30, PE-pGO membranes, and PE-iGO membranes, Figure S3: (**a**) The impact of operating pressure on DR80 rejection for commercial UF030, UF100, UF150 membranes (Microdyn Nadir). (**b**) Photos of filtrates by UF30 and PE-iGO at different pressure (1, 2, 3, 4, 5 bar).
