# Supplementary material for: Functionalized Graphene Oxide Modified Polyethersulfone Membranes for Low-Pressure Anionic Dye/Salt Fractionation
Source: Polymers (Basel). 2018 Jul 19;10(7):795. doi: 10.3390/polym10070795 (PMC6403891; doi:10.3390/polym10070795)
Supplement: Supplementary file 1 [file polymers-10-00795-s001.pdf]

Supplementary materials

Article

Functionalized Graphene Oxide Modified Polyethersulfone Membranes for Low-Pressure Anionic Dye/Salt Fractionation

Lifen Liu [1], Xin Xie [1], Rahul S. Zambare [2], Antony Prince James Selvaraj [2,*], Bhuvana NIL Sowrirajalu [2], Xiaoxiao Song [1,*], Chuyang Y. Tang [3] and Congjie Gao [1]

[1] Centre for Membrane and Water Science & Technology, Ocean College, Zhejiang University of Technology, Hangzhou, 310014, China; lifenliu@zjut.edu.cn (L.L.); 2111601112@zjut.edu.cn (X.X.); gaocj@zjut.edu.cn (C.G.)

[2] Environmental and Water Technology Centre of Innovation (EWTCOI), Ngee Ann Polytechnic, 599489, Singapore; rahul22288@gmail.com (R.S.Z.); bso2@np.edu.sg (B.N.S.)

[3] The University of Hong Kong, Department of Civil Engineering, Pokfulam, Hong Kong; tangc@hku.hk

* Correspondence: songxiaoxiao@zjut.edu.cn (X.S.); jap2@np.edu.sg (A.P.J.S.); Tel.: +86-571-88324135 (X.S.)

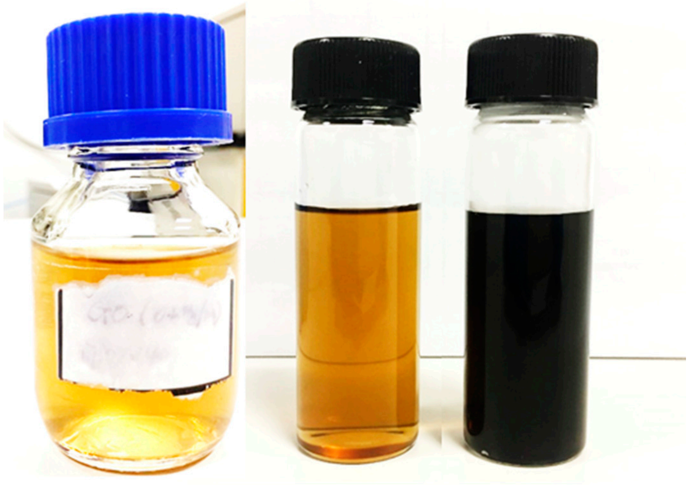

GO iGO pGO

Figure S1. The as synthesized GO, iGO and, pGO aqueous solution.

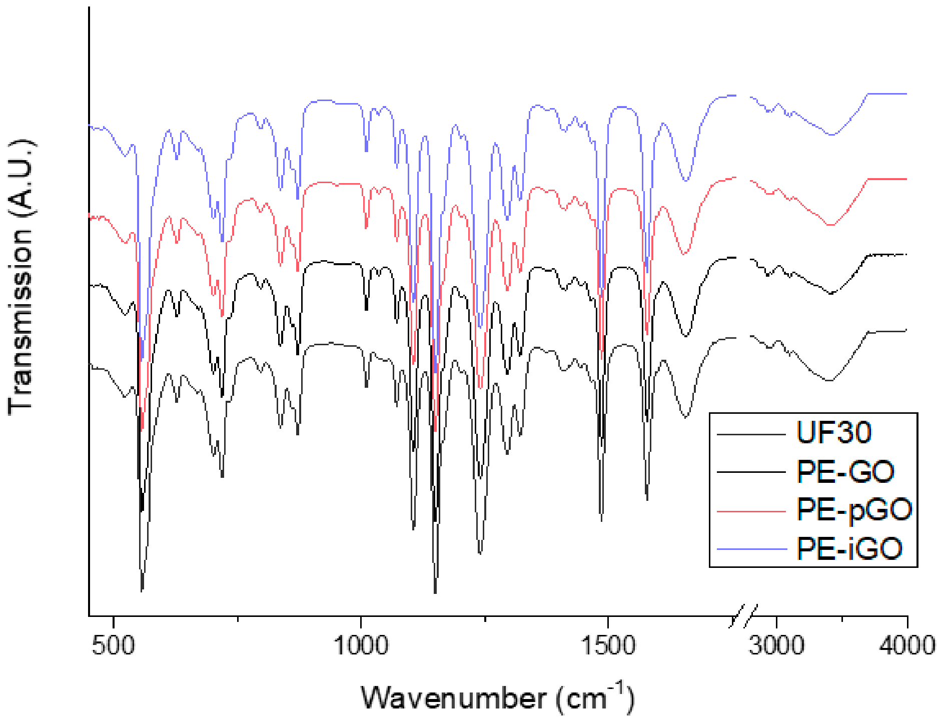

Figure S2. The ATR-FTIR absorption bands for UF30, PE-pGO membranes, and PE-iGO membranes.

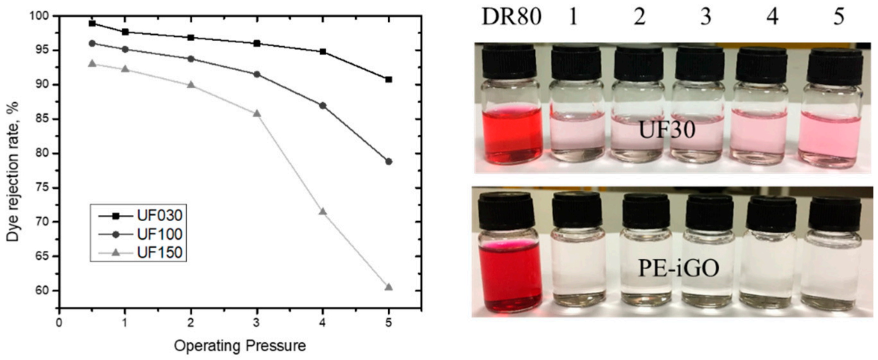

Figure S3. (a) The impact of operating pressure on DR80 rejection for commercial UF030, UF100, UF150 membranes (Microdyn Nadir). (b) Photos of filtrates by UF30 and PE-iGO at different pressure (1, 2, 3, 4, 5 bar).
